# Successful Laparoscopic Repair of Posttraumatic Diaphragmatic Injury

**DOI:** 10.7759/cureus.10071

**Published:** 2020-08-27

**Authors:** Khuram Khan, Saqib Saeed, Farhana Iqbal, Leaque Ahmed, Brian Donaldson

**Affiliations:** 1 Surgery, Columbia University College of Physicians and Surgeons, New York, USA; 2 Surgery, Harlem Hospital Center, New York, USA; 3 Internal Medicine, Richmond University Medical Center, Staten Island, USA; 4 General Surgery, Columbia University College of Physicians and Surgeons, New York, USA

**Keywords:** trauma, stab wound, diaphragmatic injury, laparoscopy, thoracoabdominal

## Abstract

Diaphragmatic injuries can be a direct result of penetrating thoracoabdominal trauma such as gunshot or stab wounds. Diaphragmatic rupture can lead to herniation of intra-abdominal organs into the thoracic cavity. Diagnosis can be difficult as the results of a physical exam can be unremarkable. A CT scan of the chest is diagnostic for diaphragmatic injuries. In most emergency cases, diaphragmatic injuries are managed with laparotomy where CT was diagnostic. We report a rare case of a 25-year-old man with right diaphragmatic injury sustained after a stab wound to the right liver managed successfully with laparoscopy.

## Introduction

Diaphragmatic injuries (DIs) are rare but life-threatening conditions, and in most cases, they can be seen with both blunt and penetrating abdominal trauma. Diaphragmatic rupture after blunt thoracoabdominal trauma is seen in about 1% to 7% of cases, and in penetrating trauma, up to 15% of the cases [1]. Of these, about 8% of the cases where a patient sustained DI will need surgical interventions [2]. Management of such injuries with laparoscopy as an alternative to laparotomy has shown excellent results both diagnostically and therapeutically.

## Case presentation

**Figure 1:**
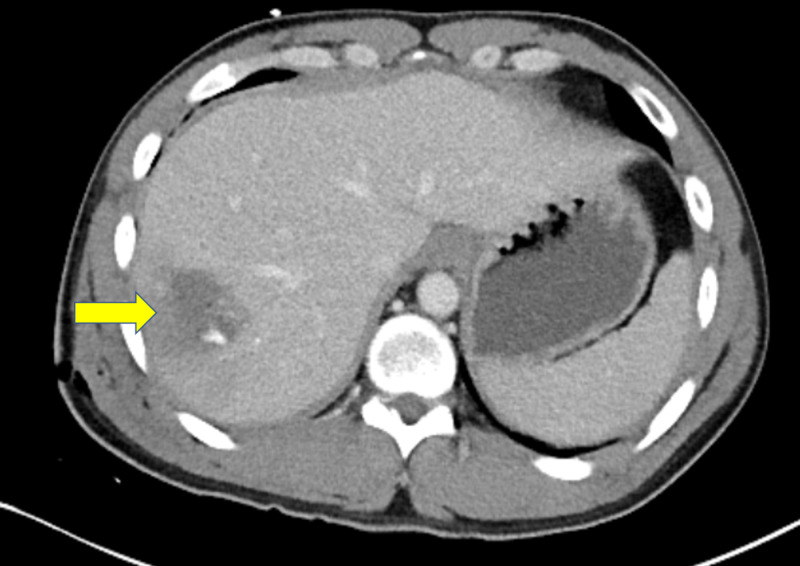
Abdomen CT, axial view, showing grade 3 hepatic laceration from knife stab wound (yellow arrow)

**Figure 2:**
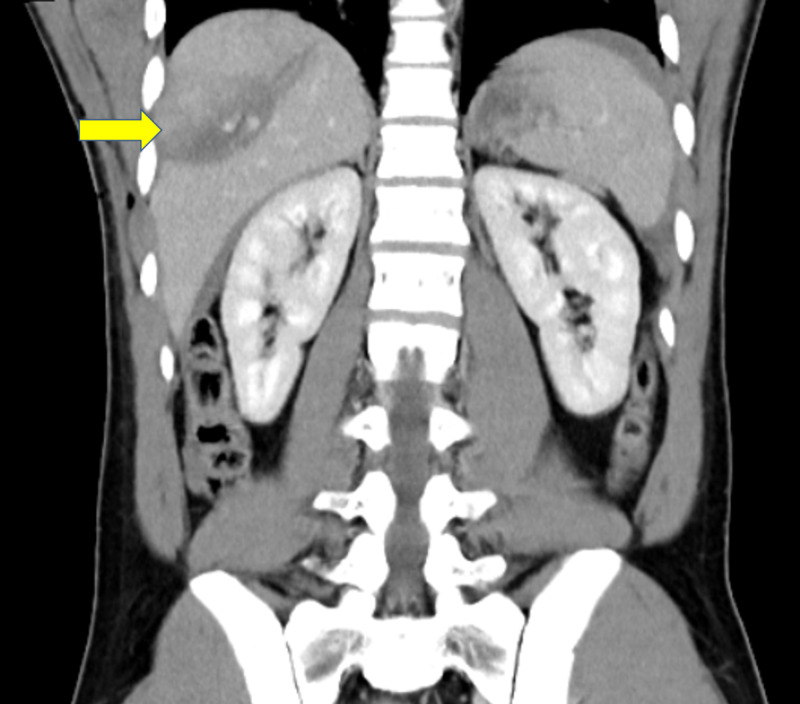
Abdomen CT, coronal view, showing grade 3 hepatic laceration after stab (yellow arrow)

A 25-year-old man with no prior medical history was transported to the hospital via the emergency medical service after he was stabbed with a seven-inch knife to the right thoracoabdominal area around the 10th intercostal space in the post axillary line. On arrival at the emergency department, he was alert, oriented, and reported right-sided abdominal pain. His vital signs were within normal limits, and oxygen saturation was 98% on room air. On physical examination, we noted a 6- to 7-cm jagged wound to the right thoracoabdominal area with profuse bleeding. The other results of his physical exam were unremarkable. No laboratory derangement was seen. Focused abdominal sonography for trauma and chest X-ray images were negative for bleeding and pneumothorax. Pressure dressing was applied to control bleeding from the wound. CT of the abdomen was obtained that revealed grade 3 hepatic laceration and no DIs (Figures 1, 2).

**Figure 3:**
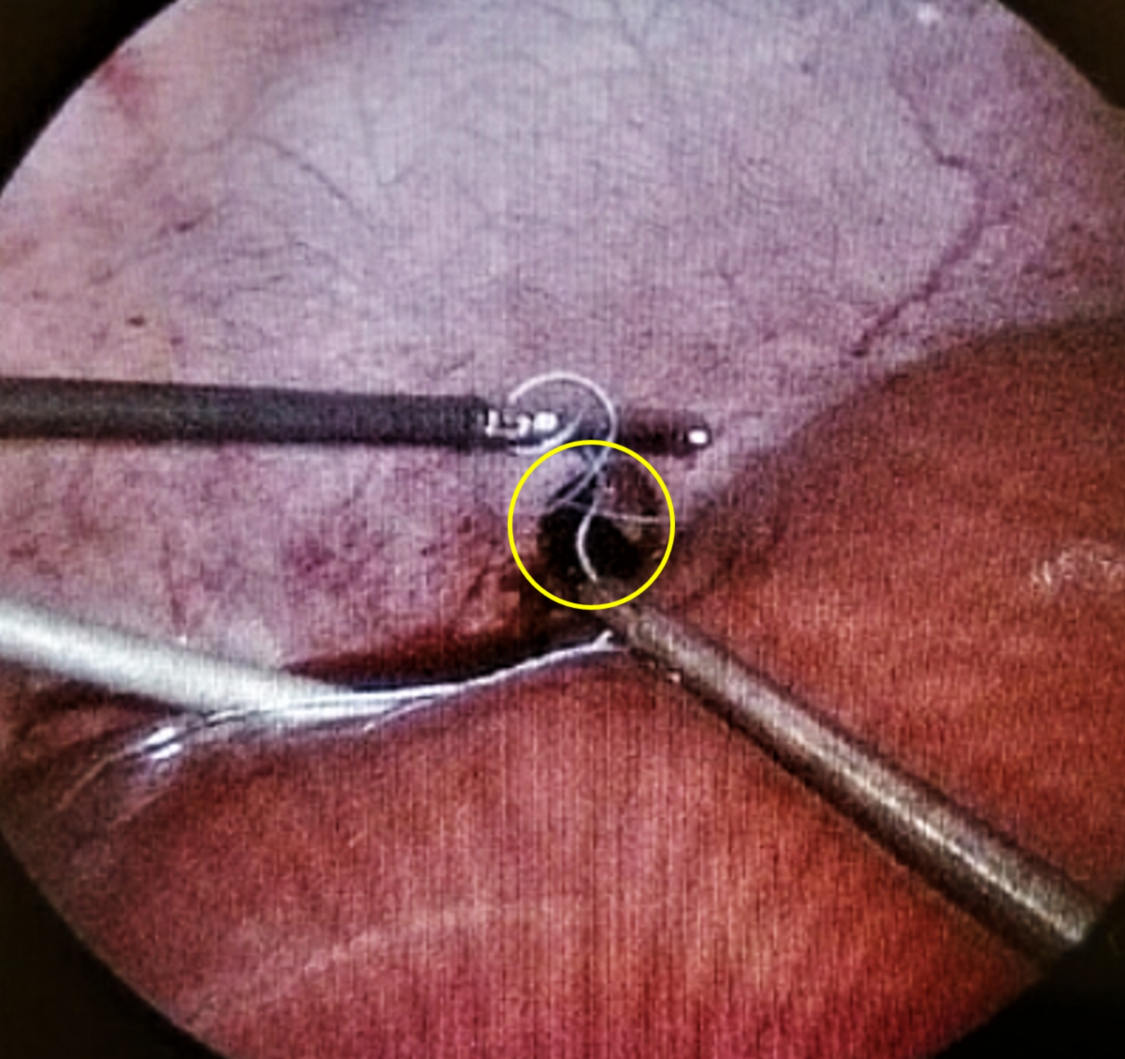
A 2-cm defect repaired via laparoscopy with suture (yellow circle outlines the defect)

**Figure 4:**
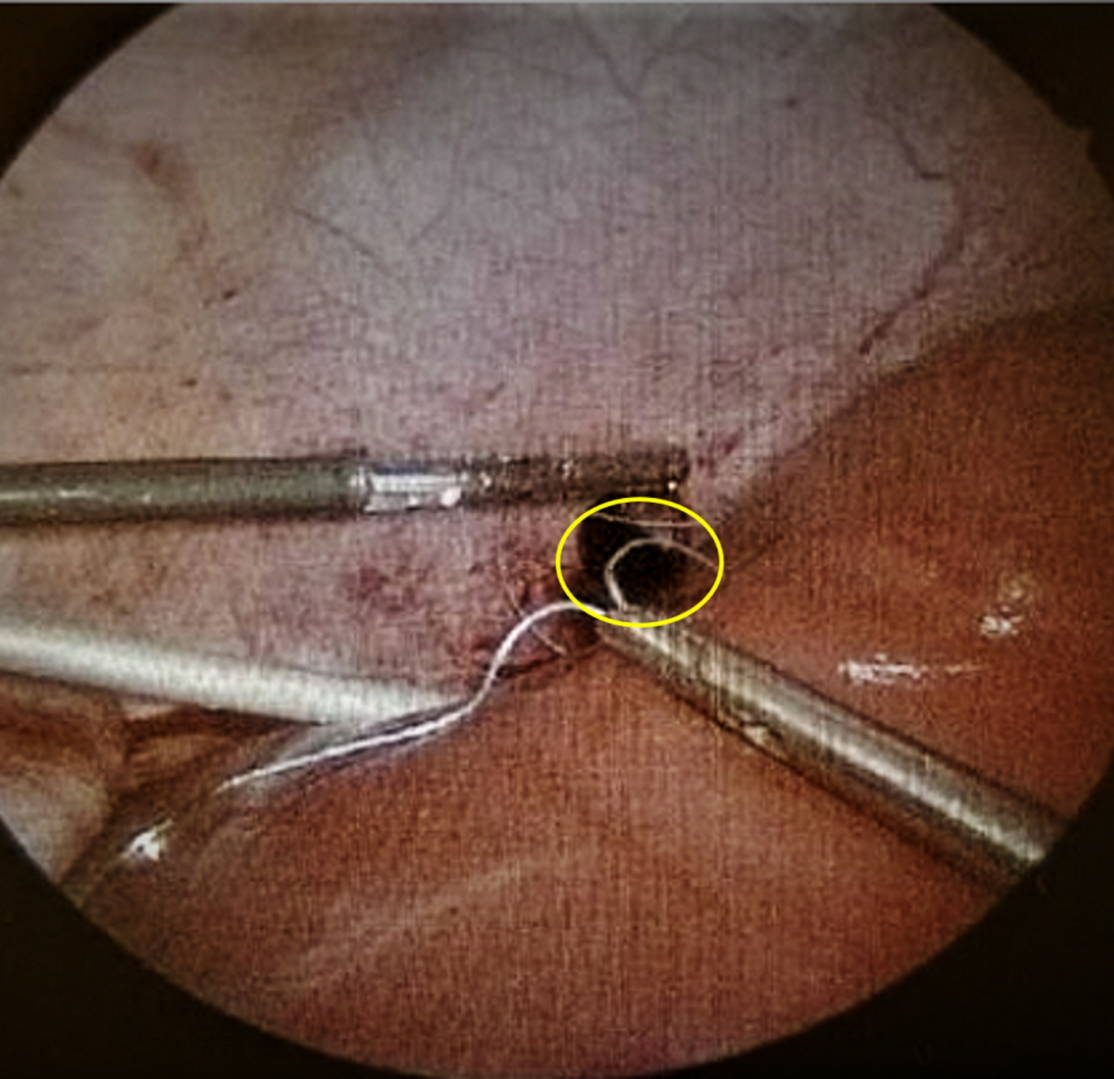
Repair of the diaphragm (yellow circle outlines defect)

He was taken to the operating room for diagnostic laparoscopy to rule out any other abdominal injuries. Intraoperatively, we noted right DI. A right-sided chest tube was placed, and the DI was sutured laparoscopically (Figures 3, 4).

Control of hepatic bleeding was achieved with hemostatic agents. The patient did well postoperatively, was extubated, and managed on the surgical floor. His chest tube was removed on postoperative day 4, and the patient was discharged to home. On follow-up visit to the clinic, the patient was doing well and asymptomatic.

## Discussion

DI is a rare and clinically underdiagnosed pathology directly resulting from blunt and penetrating thoracoabdominal trauma. Serious and life-threatening injuries can be seen with penetrating trauma and accounts for about 75% of cases [3]. One of the major challenges in successful management of a DI after trauma is diagnosing it early; correct diagnoses are often delayed, as in our case. Initial evaluation and imaging can miss most of the indicators of DI. Physical exam findings can be unremarkable, and imaging does not detect the injury that can be missed for months and even years after the initial injury [3].

In such cases, the initial evaluation in a hemodynamically stable patient can be done by obtaining a chest X-ray followed by a CT scan of the chest and abdomen. CT scans can miss 30% to 50% of the diaphragmatic rupture during the initial assessment [4,5]. Interventions such as thoracoscopic and laparoscopic have sensitivity and specificity close to 100% for DIs. Choosing thoracoscopy versus laparoscopy depends on the skillset, stability of the patient, and indications for the surgery, e.g. retained hemothorax versus suspected intra-abdominal injury. Laparoscopy allows for a broader exploration of the entire diaphragm and peritoneal cavity. Laparoscopy also offers better intraoperative visualization of the diaphragm, which can contribute to better postoperative outcomes for the patient via reduction of pain, fewer respiratory complications, and reduced length of hospital stay [5]. DIs that are seen on left side of the diaphragm should undergo repair as the right diaphragm is well protected by the liver, but in rare case like in our patient you need to repair right side diaphragm injuries. Most DI cases can undergo primary repair where large defects require placement of a mesh.

## Conclusions

Traumatic DIs can be life threatening and can be missed easily on initial evaluation after thoracoabdominal trauma. A high index of suspicion should prompt consideration of diagnostic laparoscopy to evaluate the diaphragm thoroughly in thoracoabdominal stab wounds.
